# The Preliminary Education of the Dental Student

**Published:** 1898-01

**Authors:** C. G. Kuhn

**Affiliations:** Ligonier, Ind.


### The Preliminary Education of the Dental Student.

BY C. G. KUHN, LIGONIER, IND.

What are the requirements of the dental schools in the way of a preliminary education? One would judge from reading their catalogues that it was a good common-school education. But what do they consider constitutes such an education? Have all the colleges that belong to the American Dental Association the same idea which constitutes that requirement, or do such schools construe it as their pecuniary interest may appear, as each applicant presents himself for admission?

I am willing to give the colleges credit for giving better instruction and making better professional men out of their students than they did a few years back. But if they draw the lines a little closer in regard to the student's intellectual qualifications that apply for admission, would not the men they turn out be better professional men?

I think there is not one present but will agree that the student with a college or university education is better prepared to grasp the demonstrations and lectures given by the faculty than he whose qualifications would hardly pass him out of the grammar school.

The greatest requisite of the student before entering on his professional studies is a broad, solid foundation upon which to build his profession structure, which consists of the great underlying principles of all science, truth and culture.

His mind will be so developed by the requirements of such a foundation that he will be able to arrive at a reasonably correct conclusion and capable of ascertaining the great lessons and facts from the many suppositions and demonstrations of advanced thinkers. Upon such a foundation he may be able to build a professional structure that will be an honor to the profession and himself.

It is true that upon a poor foundation he may rear a structure, and cover it with colors that glitter and deceive many, but rarely does such a structure reach completion before the trials of professional life come and carry it away to give place to one grounded and reared upon true principles of success. Perchance here and there one may stand and make what men call success, but his success is attributable more to the ignorance of the masses around him than his own merits. Some of the fathers of our profession began on poor foundations, but during practice they have been continually repairing the beginning. Dr. C. N. Johnson, in a paper read before the International Dental Congress in Paris, 1889, said, "that the first of all the evils is the allowing applicants to matriculate without the proper qualifications." There is a great laxity in this matter in most of our schools, and to it is largely due many of the ills which to-day we are called upon to criticise. It may be accepted as a rule that unless an applicant has the fundamental principles of a general education perfectly engrafted in his mind, it is impossible for a dental college to make of him a practitioner who will take his proper place in the world as a fitting representative of a learned profession, and if in future the profession is to assume the position in the community of a cultivated body of men, he must see to it that those who enter our ranks are fitted to sustain its reputation. It is true that many enter the college under the disadvantage of a limited education and by close application and a natural talent eventually make a creditable showing, but this is no argument in favor of the lax method of matriculation. The same student entering college with a good education would have advanced to a higher state of perfection than is possible without it; and it is the desideratum in all college instruction to raise the student to the highest perfection possible. The time has come when it should be rendered impossible for a young man to chose dentistry simply from the fact that he is unfitted for any other reputable profession.

It may appear strange to some of you that I have assumed this position, but, when one looks at the dental profession and sees the number that are practicing quackery, one is apt to ask the question, "Why is all this so?" For my part, I cannot help coming to the conclusion that a greater part is caused by persons whose preliminary education before entering the dental college was neglected. Call to your mind all the persons that are conducting their practice in this unprofessional manner and see what kind of an education they have, and I am convinced that seventy-five percent come under this class. Now I don't say that all persons whose preliminary education was neglected are quacks. Far from it. Many of our leading men in the profession to-day had not the advantage of a college education. No matter what the condition may have been in the past, it now lies within the means of every youth who has the mental and physical ability to become a dentist—the advantages of a good high school education.

Dr. Smith, Dean of the College of Dental Surgery in Cincinnati, Ohio, said that graduates in theology showed only twenty-five percent who had degrees in letters or any other kind of degree. In law fifteen percent, in medicine five percent, and in dentistry about the same as medicine. And that in what are now called the newer professions, engineering, etc., it was found a larger proportion had degrees than in the learned professions.

When shall we expect it to be different? I am afraid hardly so long as the different schools are making such strife to see who shall have the larger number of matriculations. Dr. G. V. Black, said, "it certainly will not while the qualifications of the pupil both for entrance and exit are controlled by that circular god with the eagle stamped on the reverse side." Gentlemen, the condition of things is to be greatly regretted, but I am glad to see at least one college—the University of Michigan—had the conviction to demand of its students that apply at their door for admission, that they have such qualifications as tend to impress the learner with the importance of a thorough comprehension of the underlying laws and principles on which alone can a solid superstructure be erected.

So, gentlemen, I conclude by saying that I think we cannot have the spirit of ethics which is sure to be stamped into our character by that training the college gives, too strongly diffused in our midst, and while the mere attending college will not make an ethical man out of one who by instinct is unethical, yet the high tenor of thought assimilated by such association cannot fall short of having an elevating effect on the man who is capable of being elevated.

